# Taxes and Tariffs

**Published:** 1895-04-20

**Authors:** 


### Modern Sociology.

### TAXES AND TARIFFS.

The Budget looms before us in the near future, and financiers and the tax-paying public alike begin to wonder on whose shoulders will be laid the burden of bearing the expense of government. The whole theory of British taxation is that the workers should go free, that no part of their earnings should be taken from them, and that no duty should be laid on the necessaries of life. Therefore our income tax is laid only on those who are proved to have already enough for these necessaries, and the tax on the money left by dead men increases with the amount. This is one method of helping the working classes, a method which perhaps they do not fully realise. They pay nothing towards the expense of the machinery of that Government which protects them, except their share of the small duty levied on tea and tobacco, and the duty on alcohol, all of them practically luxuries. In other countries heavy duties are placed on all imported articles, partly in order to raise revenue, but partly also to encourage native industry by making foreign products costly. This is more or less the case in all foreign countries, but the most familiar and convenient instance is that of the United States.

The American Republic is conveniently placed for such a policy. It has all the necessaries of life within itself. It does not depend, like Britain, on outside sources for its food supply; on the contrary, it can afford to export food-stuffs to Europe. It needs to import only manufactured goods, many of them practically luxuries; and these it is not unreasonable to tax in order to meet the cost of managing the State. But the tax is meant to do more than this. It is meant to discourage people from purchasing foreign goods, and compel them to be content with the home product. It is true that one result is to make it a matter of vanity to possess imported goods, and another to develop smuggling as a fine art, worthy to be practised by the most respectable persons. But as all cannot afford these imported things, nor have the opportunity to smuggle, the result is an undeniable development of industry. What people cannot buy they make, and thus work is found. Ample work and good pay the Protectionists have always promised, but here the other side of the question arises. "The worth of anything is so much money as 'twill bring" is only a half-truth. On the other side stands the fact that the worth of any money is just so much of anything as can be bought with it. High pay for the producer entails high prices for the product, and high prices for the product make high pay of very small value. Yet the importance of creating work for a growing population sometimes makes protection judicious. States, as well as plants and animals, require some special care in their infant years. But the principle when carried beyond infancy is harmful rather than the reverse. In Canada, which is by nature an agricultural country, the Protectionist policy has created artificial industries, quite unable to hold their own in "a fair field and no favour." Favouring them reacts to the advantage of the manufacturer, but to the disadvantage of the consumer, who has to pay a high price for an inferior article, while the energy of the people is lured into unnatural fields, and real progress is retarded rather than helped.

In the United States the industries have a better excuse for being. The American manufacturers can hold their own with the European in most fields. They require no protection from superior competition, and yet it may safely be said that protection is part of the national creed of Columbia. To a certain extent, it is true, the home product brings down the price of the imported article; but, to a much greater extent, the import duty keeps up the price of the home product. And this is the reason why the great capitalists in whom, far more than with the similar class here, political power is centred, so strenuously support it. From their point of view they are quite right. They have a larger trade and a larger business than they might have under free trade. But the object of the State should not be the producing and protecting of a handful of millionaires, but the well-being and comfort of the whole nation. Cheap goods are of as much value to the nation at large as high wages. And, moreover, the policy may be short-sighted after all. England has thriven under free trade. Wages have gone up, not down, and the purchasing power of money is greater. New industries have sprung up, made possible by the greater wealth of the community. England can support a larger population now than she did when the century began, although she buys the food which then she raised herself. Luxuries which then belonged to the few are enjoyed by nearly all. What we hear of the privations of the poor says more for the quickening of the public conscience than for any increase of want. Without being optimistic, or ignoring the flaws in our civilisation, we may hold to it that our policy has answered.

America is at present too young, too rich, has too much space for development at her command to feel as we do the need of buying in the cheapest market. It is self-sustaining, as England cannot be, and can beside export largely to meet European needs. Protection has not yet been found oppressive; after a fashion it has been profitable to the State. The heavy duties collected under the M'Kinley tariff have poured money into the Treasury, which it did not know how to use. So at least we must infer from its having been lavished in pensions, which even the firmest upholders of a fundamentally vicious political system find disgraceful. Had the tariff served only to raise the money necessary for the machinery of government no one need have grumbled; it is as convenient a way as another, and indeed the impost falls more justly than many, for the income tax is the confessed parent of an amount of lying and evasion which cannot be estimated, whereas the duty charged on tea and tobacco cannot by any means be evaded. There is nothing to be said against a moderate tariff on such articles, but when the tariff becomes a source of profit, it is time for people to ask if they have not as good a right to that profit as any pensioners of the Government.

